# Evaluation of the Fracture Resistance of Conservative and Ultraconservative Access Cavity Designs with Different Treatment Modalities: An In Vitro Study

**DOI:** 10.1155/2023/7247375

**Published:** 2023-07-13

**Authors:** Farzaneh Shirani, Masoud saatchi, Mehrangiz Shirani, Niloufar Jafari

**Affiliations:** ^1^Department of Restorative Dentistry, Dental Research Centre, Dental Research Institute, School of Dentistry, Isfahan University of Medical Sciences, Isfahan, Iran; ^2^Dental Research Centre, Department of Endodontics, Dental Research Institute, School of Dentistry, Isfahan University of Medical Sciences, Isfahan, Iran; ^3^School of Dentistry, Isfahan University of Medical Sciences, Isfahan, Iran; ^4^Department of Restorative Dentistry, School of Dentistry, Rafsanjan University of Medical Sciences, Rafsanjan, Iran

## Abstract

**Introduction:**

The aim of this study was to evaluate the fracture resistance of endodontically treated mandibular molars using traditional and conservative access cavity preparation.

**Materials and Methods:**

In this in vitro study, 100 extracted healthy human mandibular molars were selected and divided into 10 groups (*n* = 10). Healthy teeth in one group were considered the control group. In three groups, traditional access cavity preparation was done (groups A) without two marginal ridges (A1), with one marginal ridge (A2), and with two marginal ridges (A3). In three groups (group B), two separate access cavities with a dentinoenamel roof without two marginal ridges (B1), with one marginal ridge (B2), and with two marginal ridges (B3) were prepared. In three other groups (groups C), two separate access cavities were prepared only with a dentinal roof without two marginal ridges (C1), with one marginal ridge (C2), and with two marginal ridges (C3), on which root canal treatment was performed afterward. Then, these teeth were subjected to force until fracture. The fracture force and fracture mode of each tooth were recorded and compared between groups by ANOVA, Tukey's post hoc, and chi-square tests using SPSS ver. 23 (IBM, Somers, NJ, USA).

**Results:**

The control teeth had the highest mean fracture force (2804.5 ± 338.5 N), followed by a conservative access cavity with a dentinoenamel roof and two marginal ridges (2360.4 ± 181.72 N) and a conservative access cavity with a dentinoenamel roof and one marginal ridge (1812.8 ± 263.9 N), respectively. The lowest mean fracture force was found for the conventional access cavity group without two marginal ridges (399.4 ± 95.2 N).

**Conclusion:**

In the condition of this study, with two separate access cavities in mandibular molars and maintenance of the marginal ridges, it is possible to provide teeth with higher fracture resistance against occlusal forces.

## 1. Introduction

After root canal treatment, each tooth needs restorative treatment to prevent leakage and restore its function in the oral cavity. The success of any root canal treatment is closely tied to the quality of the restoration, i.e., its coronal seal. The type of restorative treatment used for these teeth depends on the amount and shape of the tooth structure remaining after endodontic treatment [1]. Preserving the tooth structure from a biomimetic point of view helps maintain the cohesion of the tooth and its biological and mechanical properties and creates a more suitable substrate for the use of adhesive restorations. On the other hand, it will increase the lifespan of the restoration and the tooth [2]. Preserving the tooth structure as much as possible increases its fracture resistance [3] and disrupts the restoration cycle [4]. During the access cavity preparation for endodontic treatment by traditional endodontic cavity preparation (TAC), large amounts of dental tissue, including the cusps, ridges, and pulp chamber roof, are lost, which increases the risk of tooth fracture after restoration [4]. On the other hand, studies have shown that only complete cusp coverage can provide the required protection for the endodontically treated teeth, which requires the removal of large amounts of dental tissue to provide a sufficiently durable form [5–7].

Some studies have introduced more conservative and ultraconservative designs for access cavity preparation [8, 9], i.e., conservative, Ninja, and truss access. The Ninja is gained through the central fossa or deepest part of the occlusal surface [10, 11]. Through this small hole, all the canals should be accessible. The truss access, or “orifice-directed access,” is in which the access targets only the canal orifices, and the dentinal bridge between the mesial and distal canals (in mandibular molars) or the buccal and palatal canals (in maxillary molars) is preserved [12, 13]. In these methods, the pulp chamber roof is not removed [6, 14, 15], and a small cavity is designed on the occlusal surface to access the orifice of the canals.

However, studies have reported contradictory results about the efficiency of using these designs as well as the effect of these methods compared to the TAC method on tooth fracture resistance [16, 17]. In addition, clinicians sometimes encounter many cases of endodontic treatment in the clinic where the tooth has already had restorations or some decay patterns that traditional and conservative access cavity preparation cannot be achieved. In these conditions, traditional design removes a large amount of remaining tissue; in addition, conservative and ultraconservative designs cannot be used in the way that studies have mentioned. Therefore, the present study was performed to introduce some new conservative designs for access cavity preparations depending on common decay patterns in mandibular molars and also evaluate the fracture resistance of endodontically treated mandibular molars with these access cavity preparation designs. The null hypothesis of this study was that there would be no significant difference in the fracture resistance rate of healthy teeth between traditional and these conservative access cavity preparation designs.

## 2. Materials and Methods

In this in vitro study, some extracted human third mandibular molars that were previously extracted in clinics (for orthodontic reasons or periodontal disease or whom candidate of full mouth dentures) were collected. The inclusion criteria were the intact teeth with clear and large pulp chamber. The size of the teeth in each group was homogenized; the exclusion criteria were any previous cracks, fractures, or caries. This study was ethically approved with the ethical approval number IR.MUI.REC.1395.3.583 (no. 395583). After clinical and radiographic examinations, 100 teeth were selected. Any remnants of soft tissue and calculus were removed from the tooth surface by an ultrasonic scaler, and teeth were then kept in 0.2% thymol solution for 24 hours to disinfect. Buccolingual and mesiodistal dimensions of the teeth were measured on the occlusal surface by a digital caliper (Mitutoyo, Suzano, Japan). The teeth were divided into 10 groups of 10 each. The number of mandibular first and second molars and third, as well as the size of the teeth in each group, was homogenized between groups to eliminate the size and shape variables, which may affect the results.

The study groups were as follows (Figures 1–9):

Control: intact mandibular molars.

**Figure 1:**
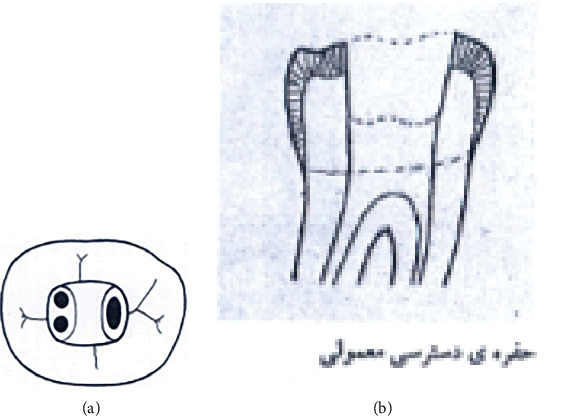
(a, b) Traditional endodontic access cavity (TAC).

Group A1. TAC: mandibular molars with traditional endodontic access cavity (Figures 1(a) and 1(b)).

**Figure 2:**
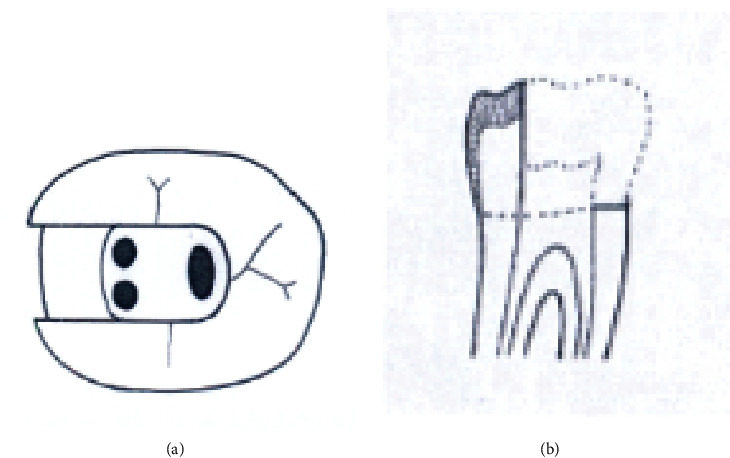
(a, b) TAC+3walls.

Group A2. TAC+3walls: traditional access cavity with a removed mesial marginal ridge (Figures 2(a) and 2(b)).

**Figure 3:**
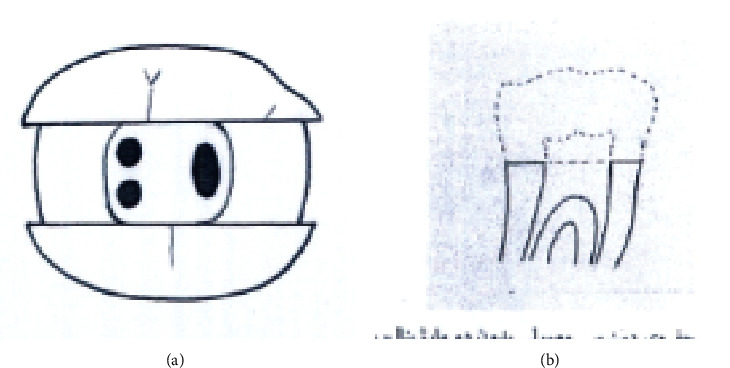
(a, b) TAC+2walls.

Group A3. TAC+2walls: traditional access cavity with both mesial and distal marginal ridges removed (Figures 3(a) and 3(b)).

**Figure 4:**
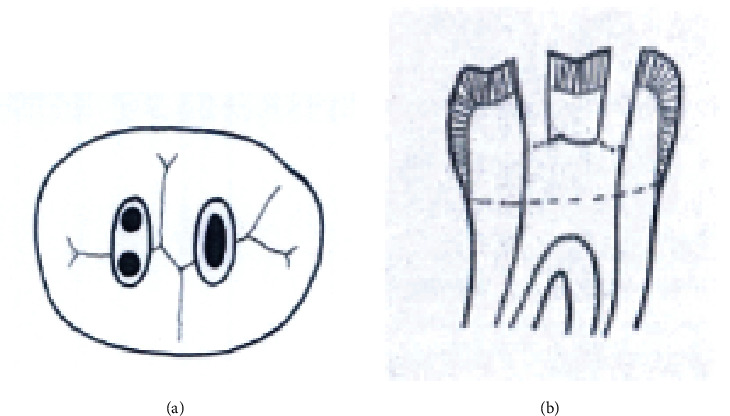
(a, b) Conservative endodontics access cavity.

Group B1. Conservative access cavity (Figures 4(a) and 4(b)).

**Figure 5:**
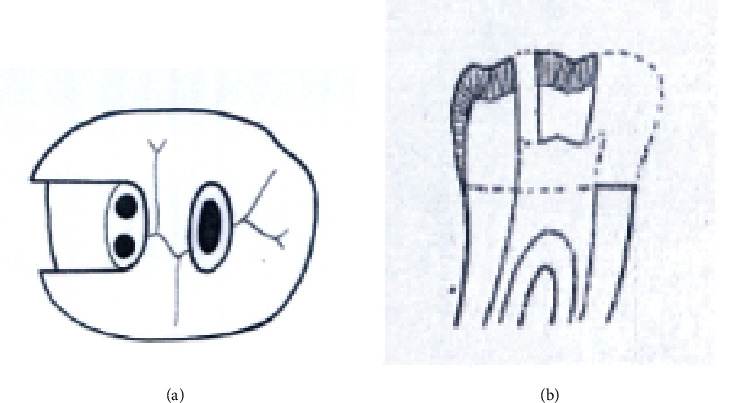
(a, b) Conservative marginal access cavity with a removed mesial ridge.

Group B2. Conservative access cavity +3walls: conservative marginal access cavity with a removed mesial ridge (Figures 5(a) and 5(b)).

**Figure 6:**
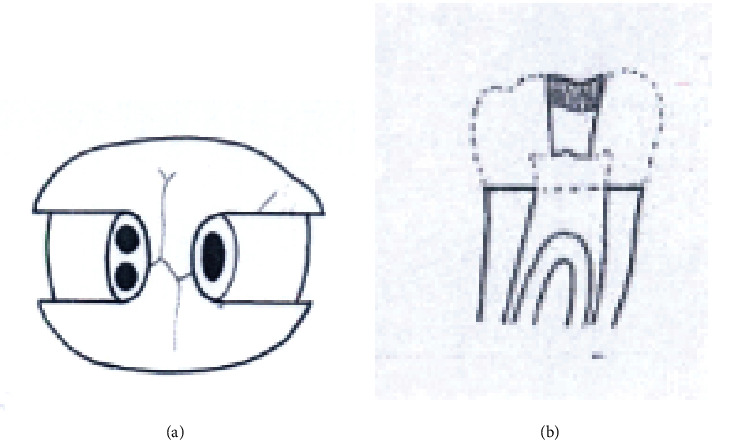
(a, b) Conservative access cavity with both mesial and distal marginal ridges removed.

Group B3. Conservative access cavity +2walls: conservative access cavity with both mesial and distal marginal ridges removed (Figures 6(a) and 6(b)).

**Figure 7:**
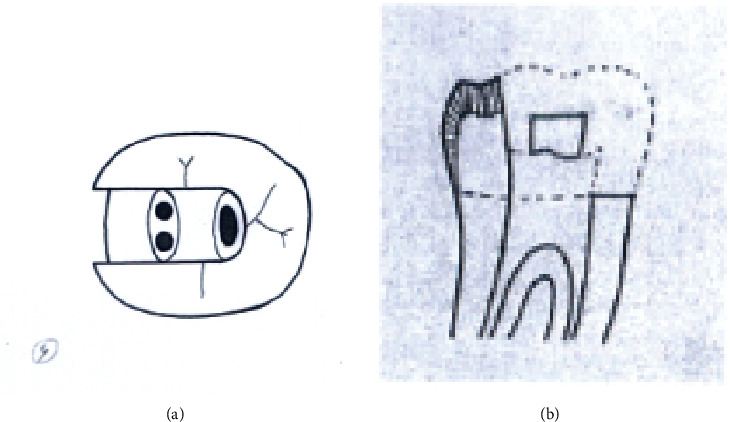
(a, b) Conservative access cavity with the marginal ridge and enamel roof removed.

Group C1. Conservative access cavity +3walls with just dentinal roof: conservative access cavity with the marginal ridge and enamel roof removed (Figures 7(a) and 7(b)).

**Figure 8:**
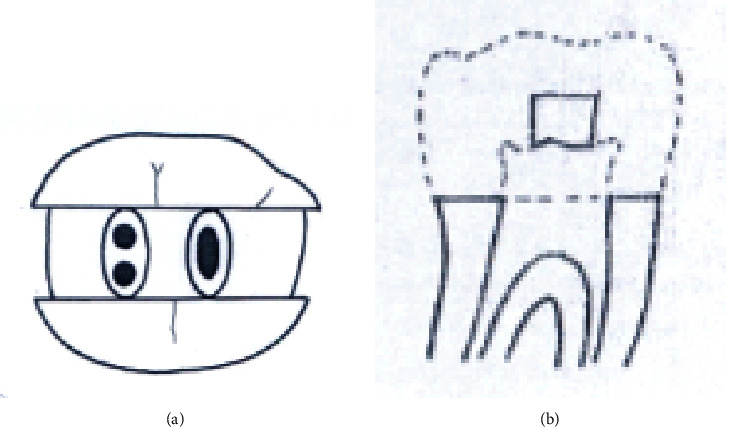
(a, b) Conservative access cavity with both marginal ridges and enamel roof removed.

Group C2. Conservative access cavity +2wall group with just dentinal roof: conservative access cavity with both marginal ridges and enamel roof removed (Figures 8(a) and 8(b)).

**Figure 9:**
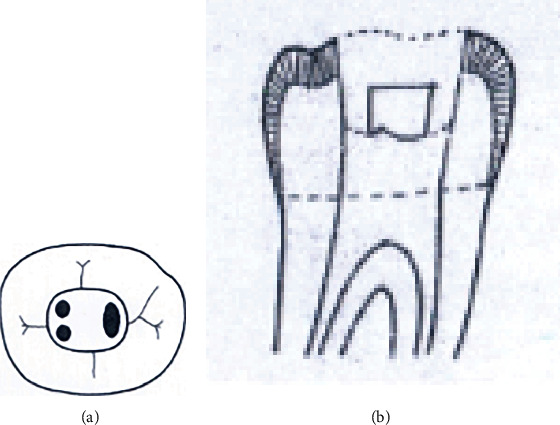
(a, b) Conservative access cavity with both marginal ridges and dentinal roof.

Group C3. Conservative access cavity + dentinal roof: conservative access cavity with both marginal ridges and dentinal roof (Figures 9(a) and 9(b)).

Access cavity preparation was done by a diamond bur no. 245 (Tizkavan, Tehran, Iran) under water-air cooling. After each tooth preparation, the bur was replaced to ensure cutting efficiency. In the TAC groups (group nos. A1, A2, and A3), the access cavity was prepared according to the principles (initial preparation of the pulp chamber was done with a diamond bur, then removal of the roof of the pulp chamber and coronal pulp tissue was done, and in the end, creating straight line access was performed) [18, 19]. In the removed marginal ridge groups, the proximal box with a width of one-half of the proximal surface was prepared, and the access cavity was connected to the proximal box.

In conservative access cavity groups (group nos. B1, B2, B3, C1, C2, and C3), to access the canals with the help of radiographs taken from the samples and to determine the distance of the canal orifice from the external tooth surfaces, the dental tissue was removed by a progressive diamond bur from the pulp chamber roof only in the upper areas of the orifice perpendicular to the tooth surface, and a slight expansion was created exactly in the form of a circle and an oval on the orifices. To remove the marginal ridge in these groups, the proximal box was created up to the cementoenamel junction (CEJ) with a width of 2/3 of the intercuspal distance and was connected to the created access cavities.

In the conservative access cavity + dentinal roof groups (group nos. C1, C2, and C3), a class I cavity with a depth of 2 mm was first created to remove the occlusal enamel, and then access to the orifice of the canals beyond the remaining dentin was created similar to that of the conservative access cavity. Then, in the groups without one or two marginal ridges, the created access cavities were connected to the prepared proximal box, with characteristics similar to the previous groups.

For root canal treatment, after determining the working length with a k-file #15, canal preparation was performed by the Protaper gold file system (Dentsply Sirona, Johnson City, USA) according to the manufacturer's instructions. The mesial canals were prepared up to F2, and the distal canals were prepared up to F2 if they had two canals and up to F3 if they had one canal. Next, a 5.25% sodium hypochlorite solution was actively used to irrigate the canals, following which they were finally rinsed with distilled water and dried with a paper point (Coltene/Whaledent/Roeko, Langenau, Germany). Canals were obturated using Gutta-perch (Coltene/Whaledent/Roeko, Langenau, Germany) by the lateral compaction technique. After root canal treatment, the specimens were mounted in a self-curing acrylic resin (Akrpars, Marlik, Iran) so that their roots were buried in the acrylic resin up to 2 mm below CEJ and kept in physiological serum at room temperature for 24 hours. For simulating the periodontal ligament, a similar procedure according to the previous study was performed [20].

To evaluate the fracture resistance, the teeth were mounted horizontally on an electromechanical universal testing machine (K-21046, Walter + Bai, Switzerland) so that they were in full contact with the lingual slope of the buccal cusps and buccal slopes of the lingual cusps while applying the force. A wedge force was applied to separate the buccal cusps from the lingual cusps at a speed of 0.5 mm/min until the teeth were fractured. The amount of force applied to the moment of fracture was recorded for each sample. Further, the fracture mode of each sample was investigated and divided into two categories, restorable and nonrestorable. The teeth fractured at 1 mm below CEJ were considered restorable, and those fractured at >1 mm below CEJ were considered irreparable.

Data were analyzed by the SPSS software (SPSS ver. 23, IBM, Somers, NJ, USA). The one-way analysis of variance (ANOVA) and Tukey's post hoc tests were used to analyze the fracture force, and the chi-square test was used to analyze the fracture mode.

## 3. Results and Discussion

### 3.1. Results

The fracture force and mode of the study groups are presented in Table 1. The highest fracture resistance was reported for the control and conservative access cavity groups (B) compared to other groups (*p* < 0.001). The lowest fracture resistance was related to group A3 (*p* < 0.001), which was statistically significant compared to other groups except for group A2, group B3, and group C1.

The mean fracture force in TAC was not significantly different from those of group B2 (*p* = 1.000), group C2 (*p* = 0.983), and group C3 (*p* = 0.263), but was significantly different from those of the other groups. Most of the fractures were restorable in the control, groups B1, B2, B3, and C1, but nonrestorable in the other groups.

## 4. Discussion

The first and one of the most important steps to performing a successful endodontic treatment is to provide a suitable access cavity so that the cleansing and obturation processes can be performed optimally [18].

During the access cavity preparation, large amounts of dental tissue are usually removed, which reduces the tooth's strength against occlusal forces; therefore, it is necessary to make maximum effort to maintain the dental tissue during the access cavity preparation without compromising the endodontic process [21, 22]. Several studies have proposed the preservation of the pulp chamber roof and the dentin around the cervical region for maximum preservation of dental structure [8, 23].

Therefore, this study was conducted to evaluate and compare the fracture resistance of endodontically treated teeth with various types of access cavities during clinical work, including traditional (TAC) and some new conservative methods designed due to more common caries patterns and previous restorations. Due to the higher probability of fracture in endodontically treated mandibular molars, these teeth were selected for the study [20, 24].

The groups studied in this research were designed based on the clinical conditions of previous caries and restorations. For example, in the groups where the dentinal roof was preserved (groups C1, C2, and C3), the tooth was clinically simulated to have a class I amalgam restoration and on one of the mesial or distal surfaces, pulpal exposure occurred, or the tooth underwent mesioocclusal restoration and exposure occurred from the distal surface, or the tooth had a mesio-occluso-distal restoration and needed root canal treatment for some reasons. Under these conditions of dentinal roof preservation, it has been shown that this roof is fractured with a little force, but this fracture will not be catastrophic [20].

One of the purposes of pulp chamber preservation in the conservative access cavity methods is to distribute heavy occlusal forces during mastication before reaching the pulp chamber floor and also to preserve the dentin of the cervical region [8, 10]. In addition, maintaining the pulp chamber roof in the mesiodistal dimension with more proximal access to the canal makes it possible to have easier access to the end of the canal and preserve more dental tissue. Nowadays, with advanced radiographic methods such as CBCT, it is possible to provide easy access to the dental canals with the least error using the methods mentioned in this study and to obtain acceptable strengthening results in teeth.

The results of the present study showed the highest fracture resistance for the control group (intact teeth) [11, 20]. The fracture resistance was significantly higher in teeth with a conservative access cavity, in which the pulp chamber roof was preserved than in the TAC groups like in some new studies that show preserving coronal dentin by using a conservative endodontic cavity significantly reduced fractures or catastrophic ones [25]. This rate was also higher in the group that had a full pulp chamber roof (groups B1, B2, and B3) (enamel and dentin) than the group that had only dentin left in the pulp chamber roof (groups C1, C2, and C3) (similar to the presence of a class I cavity), so the null hypothesis of this study was rejected. The above results show that the presence of a pulp chamber roof, especially in full as a connector of buccal and lingual surfaces, i.e., strong enamel supported by healthy dentin, is highly effective in better distribution of forces and strength of endodontically treated teeth. These results were similar to the results of the study by Plotino et al. [6], which showed that the fracture resistance was significantly higher in the conservative access cavity preparation methods than in the traditional methods. However, several studies have not indicated a difference in the fracture resistance between methods [11, 20], which can be due to reasons such as different methodology and access cavity design in these studies and our study, restorability or nonrestorability of the dentin before the test, and doing or not doing aging.

Corsentino et al. [11] conducted a study on endodontically treated teeth and reported the ineffectiveness of the truss endodontic access cavity method compared to other methods (conservative methods or TAC), but the present study shows higher fracture resistance according to the pulp chamber roof. The difference could relate to differences in the methodologic design, including the use of restoration and methodologic issues related to the design of the fracture test, and also, the removal of dental tissue was more in the mesial and distal areas of the tooth in the present study than in Corsentino et al.'s study, according to the clinical conditions. However, our study performed more simulations in the research design compared to the clinical conditions of teeth that need endodontic treatment, so the results should be interpreted more precisely. It is noteworthy to claim that more mesial and distal access to the root canal is determined by caries, not the clinician's desire to remove these tissues. Corsentino et al. [11] reported the removal of most mesial and distal ridge tissues as a factor involved in reducing fracture resistance in this study, whereas in clinical conditions, tissue removal has already been done by caries, and this risk can be used as an opportunity to access dental canals while preserving the remaining tissue of the pulp chamber roof.

In the present study, the loss of both marginal ridges drastically reduced the fracture resistance of the teeth, and the lowest fracture resistance was related to TAC without both marginal ridges and then conservative without both marginal ridges and with dentinal roof only. These results were in line with the results of previous studies highlighting the importance of the marginal ridges regarding the stability of the remaining opposing walls [26, 27].

Posterior cavities with intact marginal ridges are less susceptible to serious cusp deflection and resulting cuspal fracture than those with discontinued marginal ridges [26]. As reported in studies, marginal ridge loss leads to a 46% reduction in tooth fracture resistance, and in the case of the presence of a mesio-occluso-distal cavity, tooth strength is reduced by more than 60% [11, 12, 20]. Loss of both marginal ridges causes unrestorable fractures. It can also be argued that the presence of dentin on the pulp chamber roof alone, due to the lack of enamel that is supported by healthy dentin, will not have enough stiffness to deal with occlusal forces and is not effective in adequate tooth cohesion. However, as the results of this study showed, in the group where the pulp chamber roof was fully preserved (group B3), the fracture resistance was equal to that of the groups that had both marginal ridges, and only the pulp chamber roof was partially or completely lost (groups A1 and C3).

Furthermore, the results of this study showed the strength of teeth in the case of loss of only one marginal ridge, while the preservation of the pulp chamber roof was as high as that of the teeth with both marginal ridges and the dentinal roof of the pulp chamber, so this result is similar to the results of other studies [13], and preserving dentin as much as possible is effective in increasing their fracture resistance. In the study of Abou-Elnaga et al., access cavity preparation was done on a previously prepared mesio-occluso-distal cavity and was similar to a group in the present study in which only two buccal and lingual walls and the dentinal roof of the pulp chamber were preserved. The results obtained from the present study and Abou-Elnaga et al.'s study were different, and it seems that the smaller dimensions of dental tissue removal in Abou-Elnaga et al.'s study compared to this study have shown different results [28]. In this study, dental tissue removal in the mesial and distal regions is lower than the amount removed in the clinic following caries and pulp exposure. In addition, these teeth have been evaluated by the adhesive method after restoration.

From the fracture pattern perspective, in the case of the presence of more dental tissue, most of the fracture patterns were in the form of chipped enamel and were restorable, and in the groups where the pulp chamber roof remained, the fractures were mostly restorable. However, in teeth where the pulp chamber roof was lost, even with marginal ridges, which increased the tooth fracture resistance, the fracture patterns were more nonrestorable, and the fracture line passed beyond the marginal ridges and the pulp chamber floor. This issue is very important for endodontically treated teeth because if the tooth is nonrestorable, it has to be removed [29].

According to studies, it is possible to have direct access to the canals in the conservative methods; the only limitation, however, is the difficulty of examining the pulp chamber space and clearing it [15, 30]. Yet, the active use of canal-rinsing substances alleviates this concern by dissolving the remaining tissue [31, 32].

Conservative methods need to rinse the pulp chamber with 5.25% sodium hypochlorite to remove the pulp chamber content. One of the limitations of this study is the lack of restoration of teeth after endodontic treatment and before the fracture test. However, in this study, the teeth were not restored to determine the extent of strength loss by different methods of cavitation and to find out what pattern each of the cases followed in terms of strength and fracture mode. It is evident that with the use of adhesive dentistry and the many advances made in the methods and materials, teeth with less lost tissue can be restored by direct restoration methods, and the clinical life of teeth can be increased by preserving more dental tissue. Moreover, another limitation of the above study is that mechanical and thermal aging and dynamic forces to evaluate fracture resistance are not used. On the other hand, because most failures in the oral cavity are due to fatigue, it is not possible to completely reconstruct the oral cavity condition and to completely simulate the masticatory forces in vitro, so further studies with control of the above factors for more extensive clinical trials are required to obtain more accurate results.

## 5. Conclusion

The fracture resistance rate is higher in these new conservative methods than in TAC, and the presence of the pulp chamber roof increases the fracture resistance of endodontically treated teeth.

## Figures and Tables

**Table 1:** Descriptive features of fracture force (Newton) and fracture model in different groups.

No.	Group	Number	Mean fracture force ± standard deviation	Fracture mode	
				Restorable	Nonrestorable
1	Group A3	10	399.40 ± 95.21 [3, 5–10]	2	8
2	Group C1	10	442.30 ± 148.45 [3, 5–10]	8	2
3	Group C2	10	1154.80 ± 474.37 [1, 2, 4, 8–10]	0	10
4	Group A2	10	711.22 ± 224.98 [3, 5, 7–10]	1	9
5	Group A1	10	1145.66 ± 175.64 [1, 2, 4, 8–10]	0	10
6	Group B3	10	1011.90 ± 226.72 [1, 2, 7–10]	8	2
7	Group C3	10	1460.66 ± 294.09 [1, 2, 4, 6, 9, 10]	8	2
8	Group B2	10	1812.7778 ± 263.91 [1–6, 9, 10]	8	2
9	Group B1	10	2360.40 ± 181.72 [1–8, 10]	9	1
10	Control	10	2804.33 ± 338.56 [1–9]	10	0

Different figures above the mean of each group show the number of groups that have a significant difference with the respective group in the amount of fracture force.

## Data Availability

The data that support the findings of this study are available from the corresponding authors upon reasonable request.
